# Bleomycin ElectroScleroTherapy (BEST): mechanistic parallels to electrochemotherapy, experimental models, and unresolved questions

**DOI:** 10.2478/raon-2026-0017

**Published:** 2026-03-24

**Authors:** Barbara Lisec, Maja Cemazar, Tobian Muir, Masa Omerzel, Tanja Jesenko, Bostjan Markelc, Ales Groselj, Rok Dezman, Miha Stabuc, Dimitrij Kuhelj, Gregor Sersa

**Affiliations:** Department of Experimental Oncology, Institute of Oncology Ljubljana, Ljubljana, Slovenia; Faculty of Health Sciences, University of Primorska, Izola, Slovenia; South Tees NHS Foundation Trust, Middlesbrough TS4 3BW, United Kingdom; Faculty of Medicine, University of Ljubljana, Ljubljana, Slovenia; Faculty of Health Sciences, University of Ljubljana, Ljubljana, Slovenia; Clinical Institute of Radiology, University Medical Centre Ljubljana, Ljubljana, Slovenia; Biotechnical Faculty, University of Ljubljana, Ljubljana, Slovenia; Faculty of Health Sciences, University of Novo mesto, Novo mesto, Slovenia

**Keywords:** vascular malformations, bleomycin electrosclerotherapy, BEST, electrochemotherapy

## Abstract

**Background:**

Bleomycin electrosclerotherapy (BEST) is an emerging treatment option for vascular malformations (VMs), predominantly slow-flow venous malformations, with increasing use in other types of VMs. By combining application of bleomycin with electroporation, BEST enhances intracellular drug delivery and may improve treatment efficacy while allowing the use of lower drug doses. Although clinical evidence supporting its efficacy is growing, the biological mechanisms underlying these effects remain poorly understood. Key unresolved questions include endothelial responses to BEST, what are the dominant mechanisms of vascular injury and remodeling, and how hemodynamics and abnormal vessel architecture affect bleomycin distribution, pharmacokinetics, and effective dosing within the lesion. Although the clinical effects of BEST may be similar to the vascular disrupting effect of electrochemotherapy, it remains unclear whether these vascular mechanisms are in fact the same.

**Conclusions:**

Understanding, how bleomycin is delivered, distributed, and retained within VM tissue, and how this interacts with endothelial susceptibility and electroporation efficiency, will be essential for defining optimal dosing strategies. Addressing these questions will require experimental approaches and physiologically relevant models capable of capturing the genetic, structural, and hemodynamic features of VMs. Such advances will be critical for elucidating the mechanisms of BEST and optimizing its clinical application.

## Introduction

### Overview of vascular anomalies

Vascular anomalies include a wide range of congenital and acquired conditions affecting the vascular system. They can manifest anywhere in the body, presenting a spectrum from simple and benign to complex conditions. Vascular anomalies are categorized into two main groups: tumors (proliferative neoplasms) and malformations (morphogenetic defects). Tumors can be further classified as benign, locally aggressive/borderline, or malignant. In contrast to vascular tumors, vascular malformations (VMs) are structural anomalies that persist and enlarge proportionally with somatic growth, reflecting developmental dysregulation rather than neoplastic proliferation. VMs are subdivided into simple, combined, or associated with other anomalies. Clinically, VMs are classified into slow-flow and high-flow malformations. Slow-flow malformations affect veins, capillaries, and lymphatics, whereas high-flow malformations affect arteries and arteriovenous connections.^1-3^ Reported epidemiological estimates vary substantially across studies, largely reflecting historical differences in classification, underrecognition of mild phenotypes, and delayed clinical recognition. The adoption of the International Society for the Study of Vascular Anomalies (ISSVA) classification^2^ and advances in molecular diagnostics have progressively refined prevalence estimates, which should therefore be interpreted as approximations rather than fixed population measures. The prevalence of slow-flow malformations is estimated to be around 1 case per 1000 births, with venous malformations being the most common subtype.^4^

The clinical presentation of VMs is highly variable. Depending on the lesion type, anatomical location, and extent of involvement, patients may experience symptoms that range from cosmetic concerns to life-threatening complications, including bleeding, thrombosis, and organ dysfunction. These symptoms can substantially affect daily activities and psychological well-being, often requiring repeated medical or interventional treatments.^5^

The pathophysiology of VMs has been linked to genetic mutations affecting endothelial cells (ECs), particularly mutations in the TEK receptor tyrosine kinase/Phosphatidylinositol 3-kinase (TIE2/PI3K) and mitogen-activated protein kinase (MAPK) signaling pathways, which influence cell proliferation, vascular organization, and permeability. These mutations drive abnormal endothelial behavior and disrupt vessel stability, leading to structural irregularities, that are specific to VMs.^6^ Understanding these genetic and molecular pathways has driven the development of targeted therapies aimed at restoring vascular function or selectively ablating the pathological vasculature.

### Current treatment modalities for vascular malformation (VMs)

The management of VMs has evolved substantially over the past decades. Traditional treatment options, such as surgical excision, laser therapy and sclerotherapy, remain widely used but often fall short in deep, extensive or infiltrative lesions, where complete removal or effective local ablation is difficult to achieve. Treatment selection is highly individualized and depends on the lesion type, severity and anatomical location. A range of diverse therapeutic options are available that include observation, sclerotherapy, laser therapy, embolization, and surgery.^1,2,7,8^

Sclerotherapy is a cornerstone of VM management and utilizes agents such as ethanol, sodium tetradecyl sulfate, and bleomycin to induce endothelial damage and subsequent vessel destruction. Despite its utility, conventional sclerotherapy can be limited by incomplete efficacy, recurrence, and complications, such as tissue necrosis or systemic toxicity.^9^ These limitations have motivated the search for approaches that are more targeted, predictable and safer.

Advances in understanding the genetic basis of VMs have led to the introduction of targeted therapies. Mutations within a small set of signaling pathways inspired the repurpose of molecular inhibitors initially developed for cancer therapy. Among these, sirolimus (rapamycin), an inhibitor of the PI3K/RAC-gamma serine/threonine-protein kinase/Serine/threonine-protein kinase mTOR (PI3K/AKT/mTOR) pathway, is by far the most widely used in clinical practice due to its manageable safety profile and reproducible symptomatic benefit across slow-flow VMs. Other targeted agents, including miransertib (Protein Kinase B [AKT] inhibitor), alpelisib (PIK3CA inhibitor), and trametinib (MEK inhibitor), are prescribed far less frequently and are generally restricted to genetically selected, severe cases due to more limited safety data and higher toxicity concerns.^10,11^ Importantly, with the partial exception of sirolimus and genotype-restricted use of alpelisib, these agents are still considered investigational for VMs. Ongoing studies are aimed at defining efficacy endpoints, optimal dosing, treatment duration, and long-term safety.^11^

In oncology, electrochemotherapy (ECT) showed how electroporation can enhance intracellular delivery of bleomycin. The demonstrated efficacy of ECT for treatment of tumors encouraged the adaptation of this principle for VMs, leading to development of BEST. By improving local drug uptake while reducing systemic exposure, BEST offers a more focused alternative to conventional sclerotherapy and has shown promising clinical use, resulting in improved therapeutic outcomes.^12,13^

### Emergence of electrosclerotherapy and clinical experience with BEST

BEST has gained increasing attention as a minimally invasive option for treating VMs, particularly low-flow lesions such as venous and lymphatic malformations^14-16^, and more recently extending to the treatment of arteriovenous malformations (AVMs).^17^ BEST was first reported in 2017 to enhance the local effect of reduced-dose bleomycin in a case where higher systemic exposure was contraindicated.^12^ Following this, early clinical reports and small case studies indicated that BEST could induce meaningful lesion regression, reduce symptom burden and may decrease the number of treatment sessions required compared with conventional sclerotherapy. In a retrospective observational case series by Wohlgemuth *et al*., BEST achieved a high response rate, with more than 80% of patients exhibiting partial or complete symptom resolution. Importantly, the procedure was associated with fewer complications than conventional sclerotherapy, highlighting its potential as a durable and minimally invasive treatment option for complex VMs.^18,19^

The favorable clinical outcomes observed with BEST in VMs mirror those reported for ECT in oncology, where electroporation-enhanced drug delivery contributes to effective local tumor control.^20^ These similarities have raised important questions about the underlying mechanisms that drive vascular responses in VMs. A deeper understanding of these processes will be essential for refining treatment parameters, improving patient selection, and developing more effective or synergistic therapeutic strategies.

## Purpose of the review

This review explores the biological mechanisms and clinical applications of BEST in treating VMs, with a focus on its similarities to ECT and the underlying molecular pathways that influence treatment efficacy. By examining the cellular and molecular features of VM-ECs and comparing them with tumor endothelium, we aim to shed light on the potential shared molecular therapeutic targets and responses to electroporation-based treatments. This review will also discuss the current *in vitro* and *in vivo* models used to study VMs and their response to treatments, highlighting recent advances in translational models that may bridge preclinical findings with clinical applications.

## Overview of electrosclerotherapy and electrochemotherapy (ECT): mechanisms and similarities

Both, ECT and BEST exploit pulsed electric fields to transiently increase cell membrane permeability, enabling otherwise impermeant or semi-permeant drugs to enter cells. In ECT, electric pulses are applied after systemic or local administration of a cytotoxic drug, most often bleomycin or cisplatin, which markedly increases intracellular drug uptake and cytotoxicity. For bleomycin, which is a large, hydrophilic molecule with limited passive cell membrane permeability, electroporation can increase cellular uptake by several orders of magnitude.^21,22^ Once inside the cell, bleomycin induces DNA strand breaks, leading to cell death.^23^ In BEST, the mechanism is analogous: electroporation facilitates bleomycin uptake into cells within VMs, resulting in size reduction of lesions like those seen in ECT-treated tumors, although the precise molecular pathways remain insufficiently defined.

Several studies have shown that ECT has a significant vascular disrupting effect on tumor vasculature. It was demonstrated that ECT induces a biphasic reduction in blood flow. Initially, a rapid vasoconstriction occurs due to electric pulses, followed by a longer-lasting phase where endothelial integrity is compromised, leading to a decrease in tumor perfusion, a phenomenon known as the “vascular lock”.^24^ Furthermore, ECT directly damages ECs, increasing vascular permeability and leading to vessel collapse within hours. This endothelial disruption, potentiated by the cytotoxicity of chemotherapeutic drug, leads to thrombosis and vessel destruction, ultimately resulting in sustained ischemia and necrosis of the treated area. In some studies, a complete destruction of tumor vasculature has been reported.^25,26^ Additionally, ECT induces significant blood flow changes within the tumor microcirculation, further reducing perfusion and contributing to tumor cell death.^24^

BEST is an emerging therapeutic approach that combines bleomycin administration followed by electroporation. Bleomycin is widely used as sclerosant to treat venous malformations, with its effectiveness significantly improved by the application of electric pulses.^12^ Clinical evidence indicates that BEST is associated with reduced intralesional blood flow and progressive reduction in lesion volume in patients with VMs. During follow-up imaging, functional impairment and reduction in lesion size are evaluated.^27^ These effects are most consistently reported in venous and lymphatic malformations, and more recently in AVMs using modified protocols.^28,29^ Long-term follow-up demonstrates durable lesion regression and sustained symptom improvement, with imaging findings commonly interpreted as progressive vascular sclerosis and remodeling.^30^ However, direct histological data defining the extent, composition, and temporal evolution of tissue remodeling after BEST are largely missing, and the relative contributions of fibrosis versus endothelial loss and vessel collapse remain undefined.

Despite differences in clinical application, with ECT targeting cells in tumors and BEST targeting VMs ECs; both approaches share cellular targets, and common mechanistic pathways, including DNA damage, oxidative stress, inflammatory signaling, and vascular damage.^24,31^ Bleomycin, a glycopeptide antibiotic, exerts its primary effect by inducing DNA double-strand breaks. After ECT, tumor cells exhibit extensive DNA fragmentation, leading to apoptosis or mitotic catastrophe.^32^ In contrast, the mechanisms underlying BEST are still largely based on assumption. Although clinical and imaging studies consistently show durable lesion regression and reduced vascularity^14,18,33^, the cellular and molecular responses of VM endothelium have not been directly studied. Key processes, including endothelial DNA damage, cell cycle arrest, modes of cell death, and fibrotic remodeling, are therefore assumed from experience with ECT and from bleomycin effects described in pulmonary ECs.

## Endothelial cell biology in vascular malformations (VMs) and tumors

ECs line the inner layer of all blood and lymphatic vessels and play a central role in maintaining vascular integrity. They regulate barrier function, vessel tone, and remodeling in response to mechanical forces and biochemical signals from the circulation. ECs are highly heterogeneous and adapt their phenotype based on their vascular bed: arterial, venous, capillary, or lymphatic. This diversity is essential for organ-specific vascular functions but also renders ECs susceptible to dysregulation under pathological conditions.^34^

### Endothelial cell heterogeneity in tumors and vascular malformation (VMs)

Endothelial homeostasis is regulated by mechanical forces and a complex interplay of growth factors and signaling pathways. Under pathological conditions, such as cancer and VMs, this equilibrium is disrupted, giving rise to aberrant endothelial phenotypes.

In tumors, ECs are highly heterogeneous, hyperproliferative, and structurally abnormal. They form disorganized, leaky vessels that support tumor growth, immune evasion, and resistance to therapy.^35^ Hypoxia-driven secretion of pro-angiogenic signals, particularly vascular endothelial growth factor (VEGF), is a dominant driver of this pathological angiogenesis and endothelial dysfunction.^36^

In contrast, VMs typically result from developmental or somatic mutations that impair endothelial signaling and vessel morphogenesis. ECs within VMs exhibit defective activation of pathways, such as PI3K-AKT and Neurogenic locus notch homolog protein 3 (Notch), leading to abnormal vessel dilation, instability, and frequent hemorrhage.^37^

Understanding the molecular mechanisms behind endothelial plasticity, including metabolic reprogramming, epigenetic regulation, and phenotypic switching, is essential for identifying therapeutic targets and improving vascular-targeted treatment strategies. For example, modulation of endothelial metabolism is emerging as a strategy to either enhance angiogenesis in ischemic tissues or suppress it in tumors.^36^ Moreover, single-cell transcriptomic approaches have revealed extensive endothelial heterogeneity even within the same tissue, underscoring the dynamic nature of endothelial identity in health and disease.^35^

**FIGURE 1.**
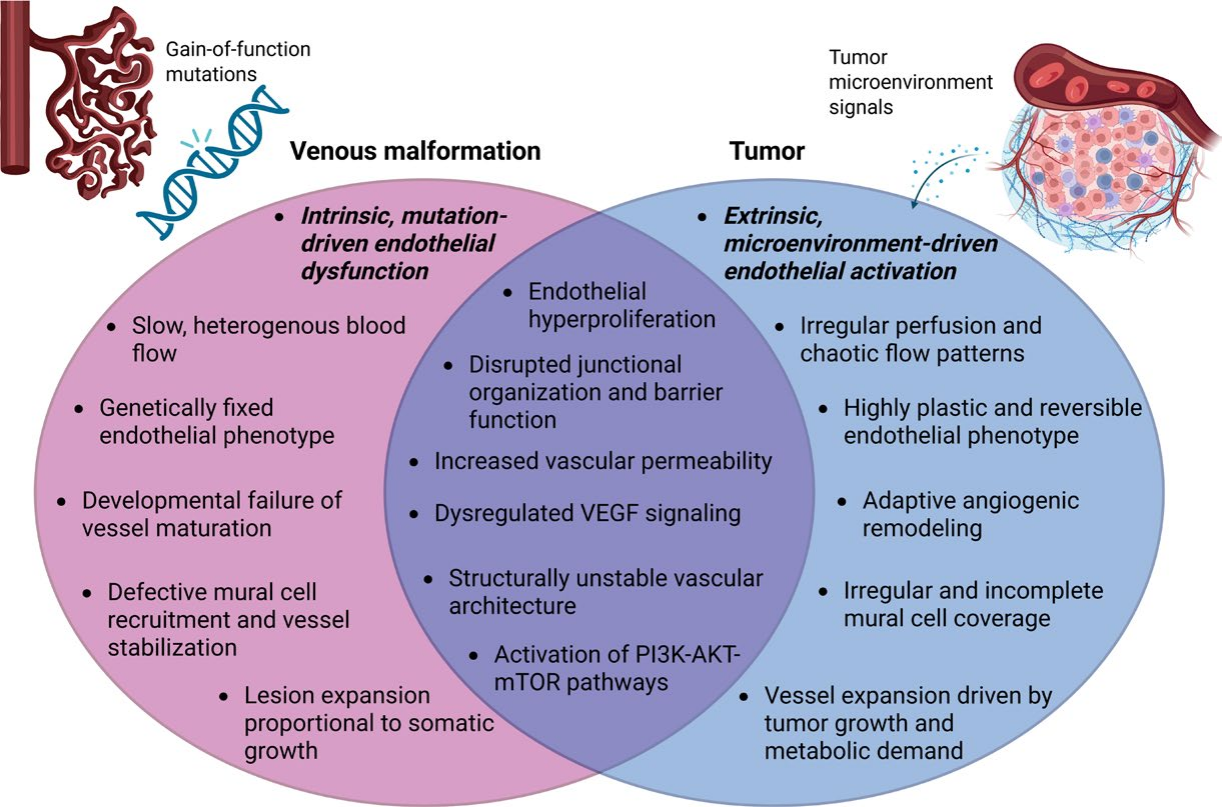
Shared and distinct endothelial phenotypes in (venous) VMs and tumors. Created in BioRender. Cemazar, M. (2026) https://BioRender.com/wo8u8ja VEGF = vascular endothelial growth factor

Together, these observations highlight that while tumors and VMs arise from distinct biological contexts, both are characterized by profound and heterogeneous endothelial disfunction (Figure 1).

### Phenotypic and functional properties of endothelial cells in vascular malformation (VMs)

Low-flow venous malformations are the most common type of VM. They are characterized by structurally and functionally abnormal EC that contribute to the formation of dilated, leaky, and poorly supported venous channels.

Human tissue analysis demonstrates that ECs in VMs exhibit abnormal endothelial differentiation and fail to establish a stable, mature venous identity. Junctional organization is disrupted, as reflected by discontinuous and punctate expression of VE-cadherin and CD31 in the majority of VM vessels, in contrast to the continuous junctional pattern observed in normal veins.^38^ This altered endothelial architecture contributes directly to vascular leakiness and mechanical instability. VM endothelium also exhibits abnormal vascular identity. In addition to venous markers, ECs frequently express arterial markers such as Delta-like protein 4 (DLL4) and Ephrin-B2, indicating a mixed or hybrid endothelial phenotype. Vascular endothelial growth factor receptor (VEGFR) expression is increased in VM vessels, consistent with heightened responsiveness to angiogenic signaling.^38^ Many vessels retain venous characteristics while simultaneously expressing arterial markers, supporting the concept of failed differentiation.

At the cellular level, ECs in VMs are hyperproliferative. A significantly increased fraction of Proliferation marker protein Ki67-positive ECs is observed within VM vessels compared with normal control veins. In parallel, VM endothelium expresses progenitor-associated markers, including Prominin-1 (CD133) and c-KIT (CD117), further supporting the immaturity and plasticity of ECs.^38^

**FIGURE 2.**
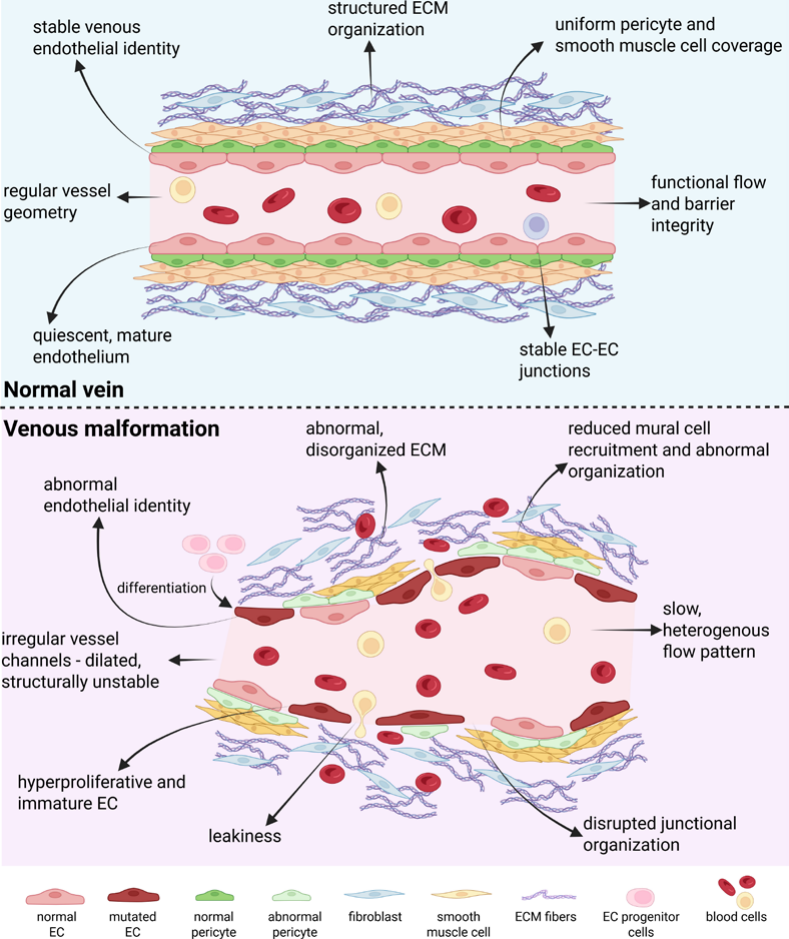
Schematic comparison of the key phenotypic and structural differences between normal venous vessels and venous malformations. Created in BioRender. Cemazar, M. (2026) https://BioRender.com/7nzewhx EC = endothelial cell; ECM = extracellular matrix

Perivascular organization is also profoundly disturbed. Instead of the orderly single-layer mural cell coverage typical of normal veins, VM vessels exhibit fragmented, discontinuous, or multilayered mural coverage. This abnormal perivascular architecture reflects defective vessel maturation and contributes to poor mechanical support and progressive vascular dilation.^39^ Ultrastructural analysis of VM-derived ECs reveals disrupted or absent basement membranes, enlarged intercellular gaps, fenestrated luminal surfaces, and aberrant vesicular profiles, all of which compromise vascular integrity and increase permeability.^38,40,41^ The key phenotypic and structural differences between normal veins and venous VM are summarized schematically in Figure 2.

At the molecular level, most venous VMs harbor somatic activating mutations in *TIE2* (*TEK*) or *PIK3CA*, converging on pathological activation of the PI3K-AKT signaling pathway.^42,43^ Most common *TIE2* mutations (e.g. *L914F, R849W)* induce ligand-independent receptor hyperphosphorylation and constitutive activation of downstream signaling cascade. This drives endothelial hyperproliferation, defective tube formation and reduced expression of Platelet-derived growth factor subunit B (PDGFB), a key regulator of pericyte and smooth muscle cell recruitment.^43,44^ Hotspot *PIK3CA* mutations (e.g., *H1047R, E542K, E545K*) define a substantial subset of VMs lacking *TIE2* mutations. These mutations similarly promote endothelial hyperproliferation, abnormal morphology, reduced pericyte coverage, and disrupted arteriovenous identity.^6^ Mouse models expressing mosaic endothelial PIK3CA mutations successfully reproduce the thin-walled, leaky vascular phenotype characteristic of human venous VMs.^40^ Notably, PIK3CA-mutant VMs display a distinct endothelial immunophenotype with high CD10 and low CD34 expression, consistent with a dedifferentiated, pro-angiogenic endothelial cell (EC) state.^45,46^

These molecular and phenotypic insights have directly prompted the exploration of targeted pathway inhibition. Both mTOR and PI3Kα-specific inhibitors (e.g., rapamycin, BYL719) have shown efficacy in restoring endothelial organization and reducing lesion size in preclinical VM models.^47^

### Shared and divergent molecular pathways in vascular malformation (VMs) and tumor endothelial cells

ECs in VMs and tumors share several pathological traits, including excessive proliferation, dysregulated angiogenic signaling, and enhanced prosurvival signaling. However, these features arise from distinct biological contexts, reflecting different modes of endothelial dysfunction.

In venous VMs, endothelial pathology is primarily mutation driven. Somatic activating mutations, most commonly in *PIK3CA* and *TIE2*, lead to constitutive activation of the PI3K-AKT pathway. This promotes endothelial hyperproliferation, impaired mural cell recruitment, and formation of dilated, slow-flow venous channels.^6^ Mislocalization of VEGF receptors and defective mechanosensing further destabilize these vessels.^42^

In contrast, tumor ECs generally lack intrinsic driver mutations. Instead, their phenotype is shaped by the cues from tumor microenvironment: hypoxia, inflammatory cytokines, and paracrine oncogenic signals reshape endothelial behavior. Tumor ECs exhibit pathological angiogenesis, vascular leakiness, and activation of survival pathways.^48^ Tumor ECs display high plasticity, resistance to apoptosis and anti-angiogenic therapy, promoting tumor persistence and immune escape.^49,50^

Despite these differences, ECs in VMs and tumors converge on a limited set of downstream signaling pathways. This shared signaling landscape underlies the growing interest in applying targeted therapies, such as PI3K or AKT inhibitors, across both cancers and VMs.^10,51-53^

Endothelial behavior in both conditions is governed by a small number of central signaling pathways that regulate growth, angiogenesis, and differentiation. Dysregulation of these pathways disrupts normal vessel structure and function, giving rise to abnormal vascular architecture observed in vascular anomalies and tumors, including hemangiomas and angiosarcomas, where angiogenesis becomes excessive and disorganized.^54^ This recognition has shifted therapeutic strategies from symptomatic management to correction of upstream molecular defects.

Vascular endothelial growth factor (VEGF) is a central regulator of pathological angiogenesis, as it drives EC proliferation, migration, and vascular permeability. In VMs, VEGFR2/3 are often mislocalized, impairing flow-dependent signaling and compromising vessel integrity.^42^ In contrast, within the tumor microenvironment, cancer cells secrete VEGF abundantly under hypoxic conditions, promoting excessive neovascularization and maintenance of a chaotic vascular network.^50,55^

Another shared pathway by VMs and tumor EC is the Notch signaling cascade, which regulates vascular architecture through its control of EC fate and vessel stabilization. Activated downstream of VEGF, Notch regulates the delicate balance between tip and stalk differentiation during sprouting angiogenesis. In VMs, aberrant Notch activity contributes to defective vessel maturation and instability.^56,57^ In tumors, Notch dysregulation supports EC survival and sustains pathological angiogenesis, reinforcing dysfunctional vasculature.^58^

**FIGURE 3.**
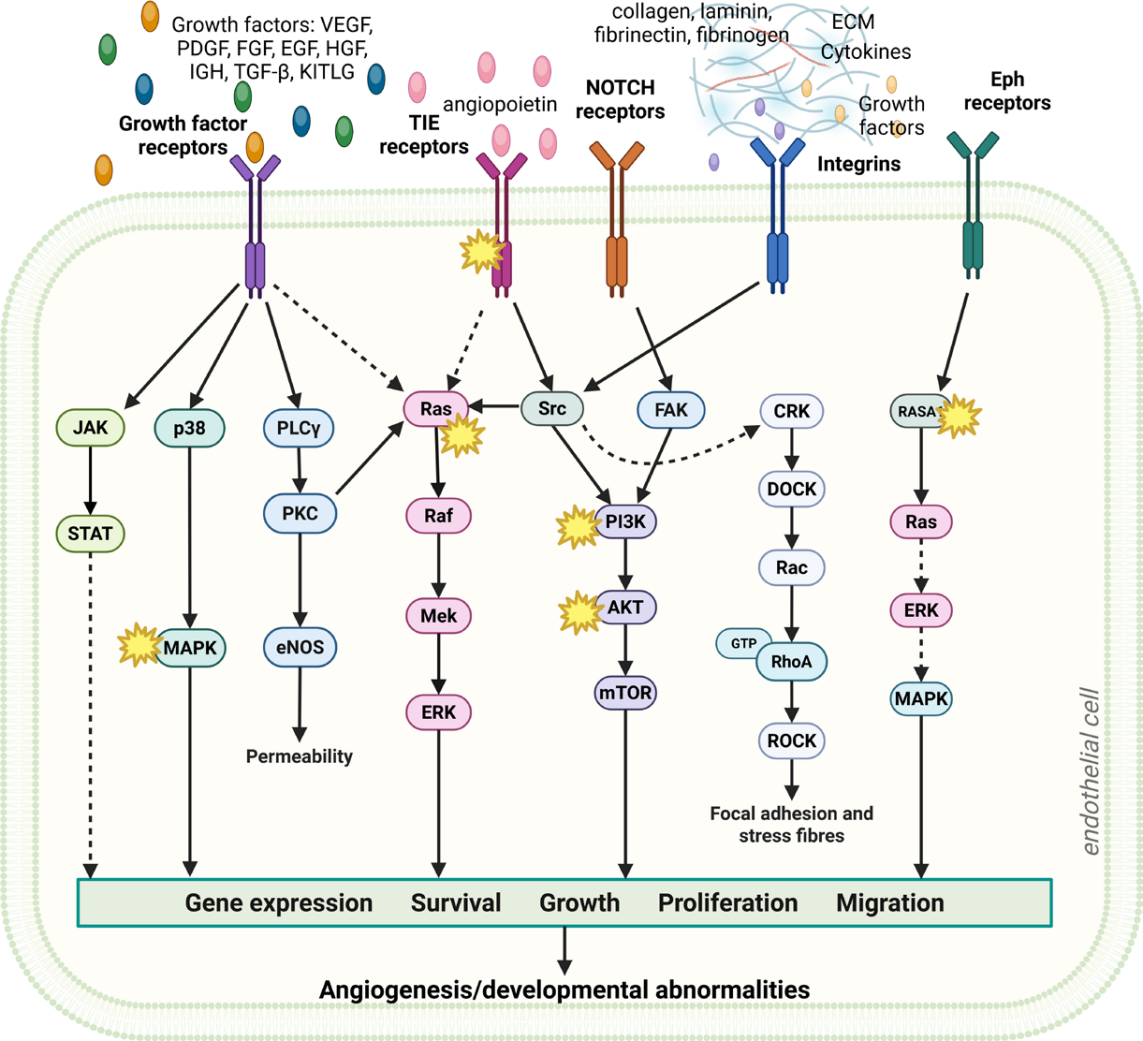
A schematic overview of shared signaling pathways in VMs and tumor angiogenesis associated with aberrant angiogenesis and/or abnormalities in vessels development and maturation. Asterisks (*) highlight mutations associated with (venous) vascular malformations (VM)s. Created in BioRender. Cemazar, M. (2026) https://BioRender.com/kfngg58 ECM = extracellular matrix; EGF = epidermal growth factor; Eph = ephrin; FGF = fibroblast growth factor; HGF = hepatocyte growth factor; IGF = insulin-like growth factor; KITLG = KIT ligant, stem cell factor; NOTCH receptors = highly conserved transmembrane proteins; PDGF = plateletderived growth factor; TGF-β = transforming growth factor-beta; TIE receptors = type of receptor tyrosine kinases that include Tie1 and Tie2; VEGF = vascular endothelial growth factor

In summary, while VM ECs and tumor ECs share common downstream signaling abnormalities (Figure 3), despite arising from distinct upstream drivers. Dysregulated VEGF, PI3K/AKT/mTOR, and Notch signaling shapes endothelial behavior and promote abnormal vessel structure in both contexts. Understanding their role is essential before drawing conclusions about how BEST or ECT affect endothelial stress responses and survival pathways.

## Therapeutic targets and molecular pathways in electrosclerotherapy

### Insights from electrochemotherapy (ECT) and implications for BEST

Bleomycin is a glycopeptide antibiotic with well-established chemotherapeutic and sclerosing properties. Its capacity to induce pulmonary fibrosis in humans and experimental animal models has made it a widely used tool for studying fibrotic vascular injury. At the cellular level, bleomycin causes single- and double-stranded DNA breaks and generates reactive oxygen species (ROS), leading to DNA damage. In pulmonary endothelial models, stress response has been linked to activation of Wnt signaling and promotion of pericyte differentiation, a process implicated in fibrotic remodeling.^59,60^

The mechanism of bleomycin-induced EC death remains complex and context dependent. Studies on pulmonary EC have shown that bleomycin rapidly triggers the extrinsic apoptotic pathway. Within 4 hours after exposure, caspase-8 is activated, followed by downstream effector caspases-3 and -6. Notably, there is no evidence for intrinsic pathway activation, as caspase-9 activation and mitochondrial cytochrome c release are absent at this early time points.^60^ Additionally, bleomycin upregulates expression of anti-apoptotic Bcl-2 family member proteins and TNF receptor genes, further supporting the dominance of the extrinsic apoptotic pathway. In parallel, bleomycin modulates endothelial inflammatory responses by upregulated expression of adhesion molecules, such as ICAM-1, VCAM-1, and E-selectin, and proinflammatory cytokines including IL-8 and MCP-1. In contrast, it does not markedly alter expression of profibrotic mediators such as TGF-β, PDGFB, or endothelin-1 in ECs.^61,62^

Beyond acute responses, bleomycin can also drive long-term phenotypic changes in ECs. Recent evidence shows that bleomycin can induce an endothelial-mesenchymal transition (EndoMT)-like program. This process involves activation of the mTOR pathway, morphological changes and altered expression of endothelial and mesenchymal markers. This process is mediated through Akt/mTOR signaling and involves transcription factors such as Slug, linking bleomycin exposure to sustained alterations in endothelial identity and function.^63^

**FIGURE 4.**
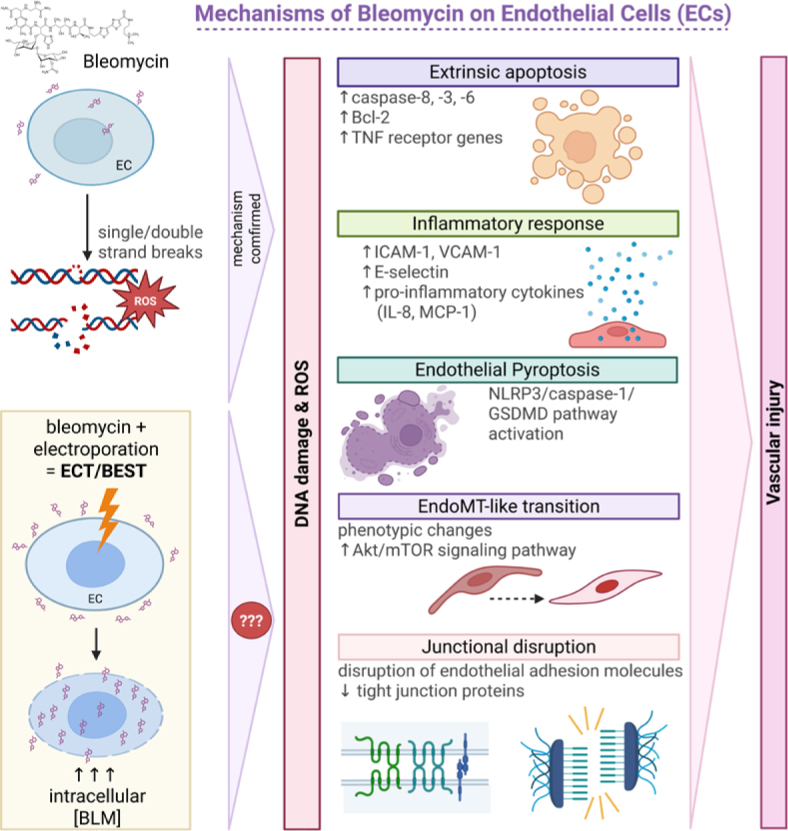
Mechanisms of bleomycin on endothelial cells (ECs). In experimental endothelial systems, bleomycin induces DNA damage, oxidative stress, inflammatory activation, apoptosis, and junctional disruption. After BEST, electroporation markedly increases intracellular bleomycin delivery, and similar injury mechanisms are presumed to happen; however, the extent and hierarchy of these mechanisms in treated vascular malformations (VMs) remain to be defined. Created in BioRender. Cemazar, M. (2026) https://BioRender.com/vyht6ij Bcl-2 = B-cell lymphoma 2; BLM = bleomycin; EndoMT = endothelial-to-mesenchymal transition; GSDMD = gasdermin D; ICAM-1 **=** intercellular adhesion molecule-1**;** IL-8 **=** interleukin 8; MCP-1 **=** monocyte chemoattractant protein-1; mTOR = mechanistic target of rapamycin; NLRP3 = NOD-like receptor protein 3; ROS = reactive oxygen species; TNF = tumor necrosis factor; VCAM-1 **=** vascular cell adhesion molecule-1

Together, these findings indicate that bleomycin affects ECs through multiple interconnected mechanisms, including DNA damage-induced stress responses, inflammatory activation, extrinsic apoptosis and EndoMT-like reprogramming (Figure 4). These effects provide a biological basis for its fibrogenic potential and support its clinical use as sclerosant, where targeted vascular injury and subsequent remodeling are desired therapeutic outcomes.

In BEST, electroporation increases the intracellular delivery of bleomycin, amplifying its effects on ECs. By transiently permeabilizing the cell membrane, electric pulses increase bleomycin uptake into ECs within VMs, thereby potentiating DNA damage and cytotoxic stress, particularly in hyperproliferative endothelium.^64^ Clinically, bleomycin is considered a relatively mild but effective sclerosant for venous VMs, and electroporation allows these effects to be achieved at much lower local drug doses.^65,66^

While endothelial injury, inflammation, and fibrosis were initially thought to explain bleomycin’s sclerosing action^67^, accumulating evidence suggests a more complex mechanism. Bleomycin disrupts endothelial adhesion molecules and cell-cell junctions in the endothelium, as well as down-regulates the expression of tight-junction proteins.^68^ A recent study demonstrated that it can also induce endothelial pyroptosis in VM tissue, an inflammatory form of programmed cell death, while simultaneously promoting fibrotic remodeling.^69^ These findings reveal that bleomycin affects several interconnected pathways, although the exact contributions of each mechanism in the context of BEST remains unclear.

The combination of bleomycin with electroporation has several advantages over conventional sclerotherapy. One study reported more than 5000-fold increase in cytotoxicity when electric pulses were applied.^70^ This targeted delivery reduces systemic toxicity and minimizes damage to surrounding tissues. In parallel, electric pulses also have a direct vascular effect, a phenomenon described as “vascular lock”, a rapid and transient cessation of blood flow following electric pulse application. In one experimental model, perfusion dropped by about 70% immediately after treatment and progressed to complete shutdown within 24 hours.^71^ Additional studies confirmed that EC damage induced by ECT disrupts vessel integrity, leading to reduced perfusion, tumor hypoxia, and amplification of the antitumor response.^24,25^ In BEST, analogous vascular responses are thought to promote vessel closure and sclerosis, leading to gradual lesion shrinkage. Clinical reports indicate meaningful reductions in lesion size and symptom improvement, particularly in venous and lymphatic malformations, where conventional treatments are often insufficient.^14-16,33^

Despite these encouraging outcomes, BEST remains an emerging treatment. Broader clinical and preclinical evidence is still needed to define its optimal application, as its mechanisms remain only partly understood. Advancing this field will depend on experimental systems that can capture the complex biology of VMs and allow direct investigation of treatment responses. This creates a clear need for reliable *in vitro* and *in vivo* models that can help uncover how BEST acts at a cellular and tissue level.

### *In vitro* and *in vivo* models for studying vascular malformation (VMs)

Understanding the mechanisms behind VMs and developing effective therapies requires robust and biologically relevant models. Because VMs are heterogeneous and structurally complex, no single model can capture all aspects of the disease. Instead, researchers must rely on a range of complimentary systems. Each of these models provides specific insights while carrying distinct limitations.

#### *In vitro* models

*In vitro* models provide controlled environments to study the molecular and cellular features of VMs. EC cultures remain an essential tool, allowing researchers to introduce VM-associated mutations to study their effects on cell proliferation, signaling cascades, junctional integrity and vessel-like structures.^72^ A notable example comes from Goines *et al*. who successfully isolated ECs directly from VM lesions and identified mutations in *PIK3CA* and *TIE2*. These mutations triggered abnormal activation of the AKT and mitogen-activated protein kinase (MAPK) pathways and drove characteristic VM-like behavior *in vitro.^73^* Another promising tool for studying vascular biology and providing a platform for modeling various vascular diseases was developed using human induced pluripotent stem cell (iPSC) derived ECs.^74,75^

Conventional 2D angiogenesis assays, such as tube-formation^76^ or sprouting assays^77^, provide a straightforward method for assessing how VM-associated mutation alter endothelial behavior. They provide a simple and informative way to assess how VM-associated mutations change endothelial organization, branching, and early lumen formation. Although simplified, they can reveal how mutated ECs respond to mechanical stress, growth factors, or pharmacological inhibition. They are also practical tools for testing how exposure to some drugs affects endothelial organization and survival in controlled conditions.

Microfluidic devices, also known as “vascular chips”, provide a sophisticated 3D perfusable environment that better mimic the dynamic conditions of blood vessels compared to 2D systems. These 3D systems can be lined with genetically modified ECs to reproduce the architecture and flow characteristics seen in VMs. Aw *et al*. developed a microphysiological model using human ECs with *PIK3CA* mutations, successfully recreating features of VMs, including abnormal vessel architecture, such as abnormal lumen formation and dysregulated flow.^78-80^ Because these devices allow real-time assessment of barrier function, shear stress responses, and drug effects, they are particularly valuable for studying how exposure to targeted drugs alters endothelial behavior in a physiologically more relevant microenvironment.

Advances in 3D printing have further enabled the creation of realistic AVM phantoms. These physical models replicate complex vessel geometry and are valuable tools studying embolization techniques and for clinical training. While they do not capture cellular pathology, they offer a safe and accessible platform for understanding how devices or injected substances behave within malformed vasculature.^81^

#### *In vivo* models

Animal models remain essential for studying VM development and for evaluating how therapies such as BEST affect diseased vasculature in a physiological environment.

*Transgenic mouse models* carrying human VM-associated mutations have been particularly useful.^53^ Using conditional inducible systems like Crelox and Tet-On allows researchers to control gene expression in specific endothelial populations. These models have helped clarify how mutations disrupt vascular remodeling, angiogenesis, and endothelial downstream signaling pathways.^82,83^

*Xenograft models* provide another valuable approach to study VMs *in vivo* and to test therapeutic approaches. These systems rely on transplanting EC, either patient-derived^84^, primary human isolates, or genetically modified human cell lines^85,86^, into immunodeficient mice. These immunocompromised strains are typically used because they allow stable engraftment of human xenografts. Studies have shown that ECs isolated from patients VM lesions carrying *PIK3CA* or *TIE2* mutations can form malformed vascular structures *in vivo*, mirroring human lesions.^73,84,85,87,88^ Such models preserve human endothelial biology and allow researchers to test targeted therapies or experimental interventions like BEST in a controlled yet physiologically relevant condition.

*Syngeneic models*, widely used in oncological research, involve transplanting EC into immunocompetent mice of the same genetic background. In principle, this approach would allow investigation of disease-relevant endothelial behavior within a fully functional immune environment. However, syngeneic endothelial grafts have not been established for VMs. ECs are intrinsically immunogenic, as they express MHC class I and II molecules, costimulatory ligands, and alloantigens, and they can function as antigen-presenting cells capable of activating alloreactive T cells.^89,90^ These properties can limit stable engraftment even when the donor and recipient are from the same strain. As a result, syngeneic approaches remain largely theoretical in the VM field, but they may become feasible as endothelial engineering and immunomodulatory strategies advance.

*Allograft models*, another approach commonly used in cancer research, offer a conceptual alternative. They involve transplanting murine EC, either primary or genetically engineered to express VM-associated mutations into immunodeficient mice. Because EC engraftment is substantially more reliable in the absence of T-cell-mediated immunity^91^, allograft approaches may offer a practical route for studying disease-relevant endothelial signaling when cells are modified to express VM-associated mutations. Although no published studies have yet applied allograft models to VMs, they may provide a flexible platform for intermediate-scale studies that bridge the gap between simplified *in vitro* systems and more complex transgenic mouse models.

**FIGURE 5.**
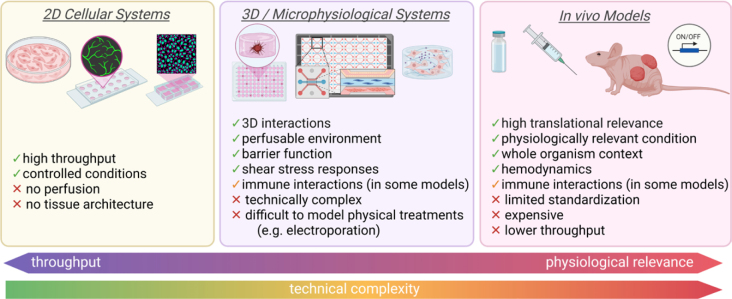
Experimental models for studying vascular malformations (VMs) and mechanisms of BEST. These models differ in complexity, throughput capacity, and physiological relevance, as well as in their ability to recapitulate native vascular structure, hemodynamics, and immune context. Simple systems are well suited for mechanistic analyses, whereas microphysiological and *in vivo* models are required to investigate flow disruption and tissue remodeling. Strategic integration of complementary model systems is essential to dissect the cellular and vascular disrupting mechanisms underlying BEST. Created in BioRender. Cemazar, M. (2026) https://BioRender.com/spmxuwa

Together, *in vivo* systems provide complementary insights into VM biology and are essential for advancing mechanistic studies and improving therapeutic strategies, including the further refinement of BEST. Figure 5 provides a schematic overview of the complementary model systems required to dissect endothelial and vascular responses to BEST.

Although recent studies have clarified several cellular effects of bleomycin and the vascular consequences of electroporation, the mechanisms driving BEST remain only partially defined. Existing models of VMs capture selected elements of VM biology but do not fully reproduce the features most relevant to electrosclerotherapy, such as altered flow, abnormal endothelial signaling, and treatment-induced vascular remodeling. These limitations restrict our ability to systematically dissect how electroporation interacts with mutated endothelial pathways or how BEST induces lesion regression. The improvement of model systems will be essential to answer the unresolved mechanistic questions raised in this review.

## Conclusions and open questions

BEST represents a promising approach for treating VMs; however, several critical aspects of its mode of action remain unresolved. Clinical outcomes after BEST appear comparable to those observed after ECT concerning the vascular disrupting effect, but direct mechanistic comparisons are lacking. This review highlights several key open questions:

- How do VM-specific mutations modify the endothelial response to electric pulses? It is unclear whether mutated signaling pathways alter membrane repair, permeability, or survival pathways following electric pulse exposure.
- What is the dominant form of EC cell death after BEST? Current hypotheses are largely extrapolated from studies on pulmonary ECs exposed to bleomycin and from vascular-disruptive effects described in ECT. However, direct investigation of endothelial responses within VM tissue after BEST is lacking.
- How do slow-flow hemodynamics and abnormal vessel architecture influence bleomycin distribution, effective dosing, and electroporation efficiency in VMs? In clinical practice, BEST produces therapeutic effects despite the use of very low bleomycin doses, but the underlying mechanisms remain unknown.

In summary, while BEST offers a rational and clinically useful treatment approach; the fundamental mechanisms that link electroporation; endothelial biology, and fibrotic remodeling remain insufficiently understood. Addressing these gaps will require model systems that better reflect the genetic, structural, and hemodynamic complexity of VMs.
